# Workflow of Healthcare Professionals Utilizing Fluorescence Imaging for Detecting Wound Bacterial Infections

**DOI:** 10.7759/cureus.85285

**Published:** 2025-06-03

**Authors:** Shaun Carpenter, Andrew Rader

**Affiliations:** 1 Department of Wound Medicine, MedCentris Wound Healing Institute, Hammond, USA; 2 Department of Podiatric Medicine, Memorial Hospital and Health Care Center, Jasper, USA

**Keywords:** bacteria, diagnosis, fluorescence imaging, infection, procedure, survey, workflow, wounds

## Abstract

Background

Many wound care specialists use fluorescence imaging (FLI) to complement the assessment of clinical signs and symptoms in the detection of wound infections, providing clinical value to patient management and healthcare economic benefits.

Objectives

To assist other wound care practices in adopting FLI technology, we addressed four key questions related to the activities essential for successful implementation: (1) Who is responsible for each activity? (2) How long does the overall imaging procedure take? (3) How much time does each activity require? (4) What patient and wound variables might influence the time needed for FLI?

Methodology

To answer these questions, surveys were collected via Qualtrics from eight physicians representing unique practices who had been trained on MolecuLight FLI (Toronto, ON, Canada) and had between 0.5 and 5 years of real-world patient experience with the technology. This study employed a cross-sectional, electronic survey design to evaluate physicians' experiences with FLI in wound care.

Results

The FLI workflow can be divided into 10 activities. In all practices, physicians are responsible for educating patients and obtaining informed consent. In most practices, physicians are responsible for all other activities related to FLI. The overall procedure requires an average of 28.8 minutes of physician time for a single wound. Patient variables affect procedure time.

Conclusions

Clinical and economic benefits, including the ability to modify wound treatment plans and reduce costs associated with managing infection-related complications, can be achieved with fluorescence imaging (FLI) to detect wound infections. This requires a relatively small time investment from physicians and is supported in some practices by nurse practitioners (NPs) and physician assistants (PAs).

## Introduction

In the United States, annually, 6.5 million people have chronic wounds [1]. Between 5% and 26% of these wounds become infected, depending on the type of wound, the severity of the wound, the size of the wound, and post-injury care received, among other factors [2]. Wounds with a higher risk of bacterial infection include surgical wounds, burns, puncture wounds, diabetic ulcers, pressure ulcers, arterial or venous ulcers, and traumatic injuries [3,4]. These infected wounds are typically managed by general surgeons, podiatrists, infectious disease specialists, plastic surgeons, and vascular surgeons with specialized training in wound care, along with allied health professionals [5].

Clinical and health economic impact of infected wounds

Wounds infected with bacteria can lead to increased pain, delayed wound healing, delays in advanced procedures such as wound reconstruction, potential complications like cellulitis, osteomyelitis, and systemic infections like sepsis for patients, and failure of cellular tissue products [6-8]. Infected wounds can also result in hospital readmissions, prolonged hospital stays, and increased treatment costs [9]. For payers, this translates to increases in the cost of chronic wound care by up to 70% [6].

Difficulties identifying infected wounds

Typical clinical signs and symptoms (CSS) of infected wounds include erythema, warmth, swelling, pain, discharge, and peri-wound edema [10,11]. Difficulties identifying infected wounds are due to the lack of specificity of signs (subjective), masked symptoms, subtle signs, and differences based on skin tone. CSS is often more apparent for acute versus chronic wounds [12].

Use of noninvasive fluorescence imaging to improve detection of wound infections

Bacteria at pathogenic loads can be visualized in real-time using fluorescence imaging (FLI) at the point of care, potentially resulting in earlier and more targeted treatment decisions [3]. This approach leverages the natural auto-fluorescence of bacteria by illuminating the wound with a safe violet light [7,13] and increases the detection of high bacterial loads compared to CSS alone [3]. Early detection facilitates timely intervention by healthcare providers and improves aspects of wound management such as cleansing, debridement, and antimicrobial intervention, preventing complications, and resulting in faster healing, while still reducing overtreatment with antibiotics [3,14,15]. Implementation of improved wound infection detection has the downstream benefit of antimicrobial stewardship, i.e., reducing microbial resistance and decreasing the spread of infection caused by multidrug-resistant organisms [14].

MolecuLight offers two handheld devices, MolecuLight i: X® and MolecuLightDX™, which have multiple FDA 510k clearances [16-18] and are the only company with commercially available FLI devices. The AMA has established two Level III Current Procedural Terminology (CPT) codes for this technology [16], which uses non-contact FLI designed to detect the presence of bacteria and biofilm in wounds greater than 104 CFU/g, defined as the Chronic Inhibitory Bacterial Load (CIBL), or the threshold of bacteria when impaired healing begins and skin substitutes, autologous grafts, and other advanced therapies fail [7,14]. With this medical technology, red or cyan fluorescence reliably indicates moderate to heavy bacterial loads. The presence of bacteria in the wound can guide wound bed preparation, sampling, and dressing selection and inform decisions regarding other antimicrobial interventions [3,15].

The objective of this study is to increase the knowledge of wound care physicians-medical doctors (MDs), doctors of osteopathic medicine (DOs), and doctors of podiatric medicine (DPMs)-regarding the step-by-step procedure for FLI of wounds. Toward that end, this study will help specialists understand the activities required for successful FLI of wounds, identify who is responsible for each step within the overall procedure, determine how much time is needed to complete each step, and recognize the patient and wound factors that can affect the time required for each activity.

## Materials and methods

An electronic physician work survey questionnaire was developed and administered via the Qualtrics platform from March 28 to April 8, 2025, to eight physicians specializing in wound care. The survey and data output for this paper were generated using Qualtrics software, January 2025 version. 

Physicians were targeted and screened-in based on specialty, title, as well as their prior training on and experience with MolecuLight devices. Physicians targeted for participation were geographically dispersed and associated with eight unique provider locations in five states and the District of Columbia. Participants were identified from an internal database of physicians who had received training on the MolecuLight FLI procedure and had performed the procedure within the past year. Invitations to participate in the electronic survey were distributed directly via email. The electronic survey methodology allowed for access to participants across geographic regions; geographic dispersion is vital for ensuring that findings are representative of a broader population and are not biased by regional influences. The survey methodology is ideal for self-assessments of processes, including an assessment of the average length of time that commonly performed activities require.

The survey was developed with assistance from nurse practitioners and clinicians experienced in performing MolecuLight procedures to ensure content relevance and accuracy of the steps involved.

To better understand the step-by-step procedure for FLI of wounds, survey participants were asked about patient education as well as about 10 separate activities comprising the Procedural Workflow for FLI. Activities included: (1) positioning the patient for image acquisition, (2) preparing the imaging system, (3) setting up image capture, (4) adjusting lighting, (5) performing fluorescence imaging (FLI) of the wound bed and peri-wound and measuring the wound, (6) using FLI to target cleaning/debridement in real time, (7) interpreting images and making a diagnosis, (8) uploading images, video, and data to the electronic medical record/electronic health record (EMR/EHR), (9) cleaning and disinfecting equipment, and (10) storing equipment.

These 10 activities represent a comprehensive breakdown of the full Procedural Workflow as identified by nurse practitioners and clinicians experienced in performing MolecuLight procedures.

For each of the aforementioned 10 activities, participants were asked to identify:

(1) Who in their practice is responsible for performing the activity

(2) The estimated amount of time each activity takes for patients with one, two, three, or four wounds

(3) How the following patient and wound variables influence the amount of time each activity requires: excess weight, mobility issues, disabilities that impact movement or flexibility, and compromised cognitive functioning

For each activity, the individual responsible for performing the activity was responsible for performing the activity was tallied.

Responses from eight physicians were used to calculate the average time for patient education and for the entire workflow. Average timings for each activity and the total workflow were calculated, along with standard deviations. The impact of wound number was also calculated to account for how each additional wound adds to the average timing of each activity. All survey data analyses were performed using the current version of Microsoft Excel (version 2504).

Physicians were asked about several variables that affect the amount of time required to complete each workflow step, including body weight, flexibility, mobility, and cognitive function. The impact of these patient and wound variables on the timing of each activity and the total workflow was calculated to determine the average impact of each variable on timing (standard deviations were also calculated).

Finally, survey participants were asked a series of questions on their wound care experiences, including common wound etiologies and the average number of wounds per patient.

## Results

Survey respondents

Responses were collected from eight physicians practicing wound care and coming from unique institutions in Illinois (*n *= 1), Indiana (*n *= 2), Maryland (*n* = 1), Pennsylvania (*n* = 2), Tennessee (*n* = 1), and DC (*n* = 1). They each cared for 20 to 200 wound patients per week and were experienced in managing pressure, diabetic, arterial, venous, trauma, and post-surgical wounds. All eight respondents treated wound patients in a variety of settings, including long-term care facilities, surgical, and acute-care settings. Respondents had 0.5 to 5.0 years of experience (mean 1.8 years) in performing FLI. Their patients had an average of 2 wounds (SD = 0.5). Among patients with multiple wounds, an average of 22.5% had them located on the same anatomical site (e.g., a single foot, leg, or surgical site; Table 1).

**Table 1:** Survey responders. ICD, International Classification of Diseases; SD, standard deviation

Initials of the responder	State	Years performing FLI	Site of FLI	Number of wound patients seen/week	Number of wounds in a typical wound patient	Common etiologies (ulcers, wounds)	For patients with >1 wound, percentage with wounds in the same anatomical area?
JJ	DC	4	Long-term care	70	2	Diabetic, pressure, venous, post-surgical, atypical	50
SL	IL	1	Long-term care	20	2	Pressure, diabetic, arterial, venous	10
DT	IN	1	Long-term care	90	3	Pressure, diabetic, arterial, and venous, trauma	20
JH	IN	1	Long-term care	200	1.5	Arterial, pressure, venous, trauma, and autoimmune	25
KH	MD	0.5	Long-term care	50	1.5	Pressure, diabetic, venous, arterial, post-surgical, traumatic	30
JK	PA	1	Long-term care	140	2	Pressure, vascular, diabetic, trauma, surgical	0
S C-Z	PA	5	Long-term care	100	2	Pressure, venous, ICD, arterial, diabetic	25
DL	TN	1	Long-term care	50	2	Pressure, vascular, diabetic	20
Average		1.8		90	2		22.5
SD		1.7		57.6	0.5		14.6

Procedure for point-of-care FLI

**Figure 1:**
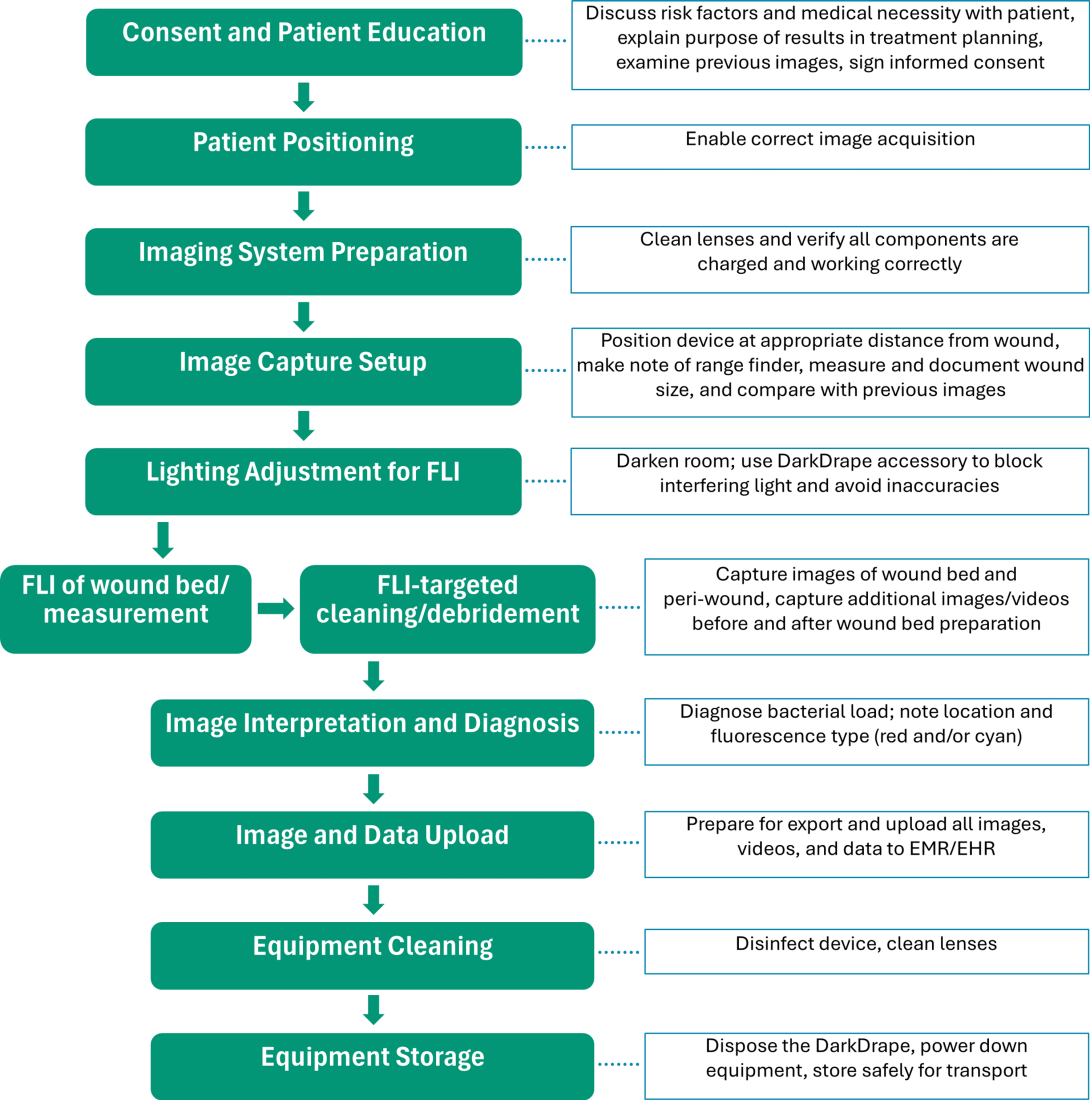
Integrated clinical workflow to deliver fluorescence imaging (FLI). Image credits: Shaun Carpenter and Andrew Rader.

The steps for the FLI workflow are shown in Figure 1. Consent and patient education (activity #1) required an average of 14.6 +/- 5.1 minutes, independent of the number of wounds to be assessed, and was typically done by the physician. The average time for the overall procedural workflow (activities #2 to #11) for a typical patient once they were educated took 28.8 (5.3) minutes, 39.5 (7.6) minutes, 52.7 (10.9) minutes, 65.3 (14.3) minutes for patients with one, two, three, or four wounds, respectively. For a single wound, the average amount of time dedicated to patient positioning (2.4 (1.6) minutes), image capture setup (4.6 (1.6) minutes), FLI of wound bed (4.0 (2.5) minutes), FLI to target cleaning and debridement (2.4 (1.5) minutes), and image interpretation and diagnosis (1.4 [1.6] minutes) increased with each additional wound (Table 2). The physician was typically the individual responsible for the actual FLI of wound bed activity and real-time wound measurements. For seven of the remaining activities (imaging system preparation, imaging system capture, lighting adjustment, image interpretation and diagnosis, uploading data to EMR/EHR, equipment cleaning, and equipment storage), the physician was deemed the responsible party by six respondents at their wound care site, with NPs/PAs responsible according to one or two respondents. Patient positioning could be done by the physician (*n* = 3) or an advanced practice provider (*n* = 4). The physician was seen as the responsible party for FLI-directed wound cleaning and debridement by five of six respondents (one site did not do this step, and one respondent did not answer). The distribution of responsibility for each activity is shown in Table 3. 

**Table 2:** Timing of each step in the FLI procedure for patients with one to four wounds. EMR/EHR, electronic medical record/electronic health record

	​Activity	Average time (minutes) (SD)					
	​		One wound​	Two wounds​	Three wounds​	Four wounds​	
#1	Consent and Patient Education ​		14.6 (5.1)	14.6 (5.1)	14.6 (5.1)	14.6 (5.1)	
#2	Patient Positioning​		2.4 (1.6)​	4.0 (2.3)	5.7 (3.4)	7.7 (4.8)​	
#3	Imaging System Preparation ​		4.1 (1.4)	4.1 (1.4)	4.1 (1.4)	4.1 (1.4)	
#4	Image Capture Setup​		4.6 (1.6)​	8.3​ (3.0)	12.5​ (4.7)	16.4 (6.1)	
#5	Lighting Adjustment for Fluorescence Imaging​		2.1 (1.2)	2.1 (1.2)	2.1 (1.2)	2.1 (1.2)	
#6	Fluorescence Imaging​		4.0​ (2.5)	6.6 (4.1)	9.9 (6.2)	13.0 (8.2)	
#7	Fluorescence Imaging for Target Cleaning/Debridement ​		2.4 (1.5)	4.2 (3.4)	6.6 (5.1)	8.9​ (6.9)	
#8	Image Interpretation and Diagnosis ​		1.4​ (1.6)	2.4 (1.9)	6.6 (5.1)	8.9 (6.9)	
#9	Uploading Images/Videos to EMR/EHR		3.0 (2.9)	3.0 (2.9)	3.0 (2.9)	3.0 (2.9)	
#10	Equipment Cleaning		2.5 (1.2)	2.5 (1.2)	2.5 (1.2)	2.5 (1.2)	
#11	Equipment Storage​		2.3 (0.5)	2.3 (0.5)	2.3 (0.5)	2.3 (0.5)	
	Total​ time		28.8 (3.7)	39.5 (3.8)	52.7 (4.4)	65.3 (5.3)	

**Table 3:** Number of respondents indicating who is the responsible party. NP, nurse practitioner; PA, physician assistant

Activity (number of responses)	Physician (MD, DO, DPM)	Advanced practice provider (NP, PA)	Did not answer	Not done
Patient Education and Consent (*n *= 8)	8	0		
Patient Positioning (*n *= 7)	4	3	1	
Image System Preparation (*n *= 8)	6	2		
Image Capture Setup (*n *= 7)	6	1	1	
Lighting Adjustment (*n *= 7)	6	1	1	
FLI of Wound Bed (*n *= 8)	7	1		
FLI-Targeted Cleaning and Debridement (*n *= 6)	5	1	1	1
Image Interpretation and Diagnosis (*n *= 8)	6	2		
Data Upload to EMR/HER (*n *= 8)	6	2		
Equipment Cleaning (*n *= 8)	6	2		
Equipment Storage (*n *= 7)	6	1	1	

Identified additional factors influencing variability in timing

Procedural workflow timing increased in patients with (1) excess body weight (body mass index > 30); (2) need for additional assistance with positioning due to age, frailty, limb loss, or hard-to-access wound sites (e.g., coccyx); (3) disabilities that impair movement (e.g., osteoarthritis, neuromuscular conditions such as Parkinson’s disease or multiple sclerosis); (4) compromised cognitive function (e.g., dementia, developmental disabilities such as Down syndrome); and (5) language barriers. In addition, patients with large wounds that could not be captured by a single image with the MolecuLight device (i.e., greater than the fluorescence field of 11.0 cm long × 8.2 cm wide for the i:X device and 18.8 cm long × 13.7 cm wide for the DX device), and those with smaller circumferential wounds that may not have fit within a single field of view, required multiple images, which added time to the procedure (Table 4). For the first five situations listed above, image capture setup, FLI, and FLI to target cleaning/debridement all took longer. For larger wounds, clinicians reported that only the FLI activity itself takes longer, with no increase in time for image capture setup or FLI to target cleaning/debridement.

**Table 4:** Factors increasing the procedural workflow timing.

Factors increasing the procedural workflow timing
First-time patients requiring additional education
Excess weight
Mobility issues (patients who require extra help moving into position)
Disabilities impacting movement or flexibility
Compromised cognitive functioning
Language barrier
Wound size (e.g., large wounds or circumferential wounds that cannot be captured by a single image)
Multiple wounds located anatomically close together
Multiple wounds located anatomically far apart
Type of wound (e.g., pressure, diabetic, arterial)

## Discussion

Clinical signs and symptoms are not sufficiently reliable to diagnose wounds with bacterial infections. CSS has poor predictive value and poor sensitivity for high levels of bacteria [3]. Multiple publications have reported that the addition of FLI to the assessment of CSS has demonstrated clinical and economic value [7,14,15,19-21].

Clinicians interested in learning how to perform FLI of wound beds are provided with training on the MolecuLight devices. There are three stages: an online self-paced eLearning including certification test (~2.5 to 3 hours), a live virtual training (1-1.5 hours), and an onsite training done over a clinic day with patients. Additional materials, including image interpretation resources, user instructions, and training videos, are provided, allowing for consistency between practices. Once training is complete, wound care specialists can perform the FLI procedure in an average of 28.8 minutes for one wound, in addition to the time needed to educate the patient and obtain consent for the procedure (average of 14.6 minutes). The amount of time needed increases incrementally if more than one wound must be imaged, since additional time is needed for each wound for patient positioning, camera setup, FLI of the wound, FLI for cleaning and debridement, and image interpretation.

Although there is some variability between practices concerning who is responsible for each activity, the survey suggests that physicians have the primary role for all activities. Proper communication between physicians and advanced practitioners is needed to provide this diagnostic service.

Patient education takes longer for a first-time patient than for a patient who has had prior FLI. Other identified patient factors that increase the amount of time needed for the procedure include patient weight, disability, mobility and flexibility, and cognitive function and type of wound, e.g., FLI would take longer for a pressure injury in a non-mobile patient. Since people over age 65 are disproportionately affected by chronic wounds and infections [22] and are often treated in long-term care facilities, many of these patient factors outlined become important considerations.

Other factors, not evaluated in this survey, that could affect the amount of time needed to complete FLI include the size of the wound (small vs. large), wound location (e.g., heel vs. coccyx), wound type (e.g., burn, post-surgical, pressure injury), and number of repeat images or videos required, i.e., if more than before and after wound bed preparation is required.

Some additional considerations that can affect how long FLI takes include whether images/videos and data will be uploaded to the EMR/EHR from the DX device or whether they are manually uploaded, which will depend on whether the physician practice can and has already had the DX device integrated with their EHR. The overall time needed for the procedure will also increase depending on the complexity of the image interpretation. Fluorescence confounders such as exposed bone and tendon (mimics cyan), excess blood (which can mask bacterial FL), and fluorescent artifacts like gauze/padding or fluorescent dyes require additional consideration when present in the images to make an accurate interpretation and diagnosis. In addition, the presence of blush red fluorescence (indicating likely subsurface bacteria) can be more challenging to determine, adding additional time to the image interpretation step. The impact of these additional variables will be assessed in follow-up research with a larger number of wound care practices.

There are several limitations to this work, including (1) the attempt to generalize results from a small number of surveyed physicians and (2) potential recall bias and subjectivity in time estimates, which may lead to inaccuracies due to self-reported times rather than direct observation or time-tracking. The goal of the survey was to identify who is responsible for each step and provide an estimate of the time taken to perform each step. While time-tracking or direct observation might be more accurate, the goal was to provide new users of the MolecuLight technology with a reasonable estimate by surveying experienced users of the technology. The relatively small standard deviations recorded for the majority of steps suggest that any errors in physician recall have some consistency. (3) The impact of variability in institutional protocols and staff roles that may influence workflow differences. Since physicians surveyed were experienced users and all received the same detailed training, there should be little to no effect of institutional protocols. Practices may vary in the level of involvement of support staff to assist the physician, but the time provided in this paper is not absolute, but rather, an estimate based on averaging eight practices.

## Conclusions

Following training with FLI devices, wound care practices can implement FLI of wounds to complement information derived from CSS. After physicians provide education to patients with respect to the procedure and obtain consent, the entire 10-activity FLI workflow process from patient positioning and imaging setup through cleanup and storage of equipment takes less than 30 minutes for a single wound. Physicians are often the responsible party for each of these activities, though in some practices, advanced practice providers can perform these activities. The inclusion of FLI into wound management can help adjust treatment plans, which can lead to improved clinical outcomes and can reduce costs to manage infection-associated complications. Wound care practitioners should be aware that various patient physical and cognitive factors, in addition to wound factors (number, size, and location), can affect the overall timing needed for successful FLI and the timing of individual activities.
